# Kidney and Pancreatic Extramedullary Relapse in Adult Acute Lymphoblastic Leukemia: A Case Report and Review of the Literature

**DOI:** 10.1155/2013/637264

**Published:** 2013-08-01

**Authors:** Leslie Skeith, Alejandro Lazo-Langner, Joy Mangel

**Affiliations:** ^1^Division of Hematology, Department of Medicine, London Health Sciences Centre, Victoria Hospital, 800 Commissioners Road East, London, ON, Canada N6A 5W9; ^2^Department of Epidemiology and Biostatistics, Western University, London, ON, Canada N6A 5C1

## Abstract

Extramedullary relapse of acute lymphoblastic leukemia (ALL) is rare and has been primarily reported in pediatric patients or hematopoietic stem cell transplant recipients. We report a case of a 62-year-old woman who presented with relapsed ALL involving her kidneys, pancreas, and bone marrow 2 years after completing chemotherapy with a standard ALL protocol. Unfortunately, her extramedullary disease progressed despite treatment. To the best of our knowledge, this is the first reported case of extramedullary relapse of B-cell ALL to the kidneys and pancreas occurring in an adult patient who had not previously undergone a hematopoietic stem cell transplant. A literature review of kidney and pancreatic extramedullary relapse in ALL is also included.

## 1. Introduction

 Acute lymphoblastic leukemia carries a poor prognosis in adults. While the complete remission rate in a patient with a standard risk profile may exceed 90% with initial treatment, the 5-year overall survival is only 30–40% [1]. Data on extramedullary relapses of ALL is limited in the adult population. The majority of the literature is in pediatric patients or in the postallogeneic hematopoietic stem cell transplantation setting. In a review of 9585 pediatric patients with ALL, 42.8% of relapses were found to be extramedullary, either with (13.5%) or without (29.3%) concomitant bone marrow involvement [2]. The most frequent sites of isolated extramedullary relapse tend to be in the “sanctuary sites” of the central nervous system (CNS) and testes, likely due to selective penetrance and reduced exposure to chemotherapy in these areas [3]. Rarely, extramedullary relapse has been reported in the ovary, breast, eye, bone, and kidneys [4–8]. Our case is the first to describe renal and pancreatic involvement of relapsed B-cell ALL in an adult patient who did not undergo a previous hematopoietic stem cell transplant. 

## 2. Case Presentation

 A 62-year-old woman presented to hospital with a one-month history of fatigue and worsening abdominal pain associated with nausea, vomiting, and early satiety. She had a previous history of precursor B acute lymphoblastic leukemia (B-cell ALL), diagnosed four years earlier when she presented with anemia (hemoglobin 72 g/L), thrombocytopenia (platelets 21 × 10^9^/L), and a total leukocyte count of 4.7 × 10^9^/L with 15% circulating lymphoblasts expressing CD10, CD19, CD20, CD34, CD79b, and HLA-DR. Her initial bone marrow biopsy revealed 80–90% lymphoblasts, medium-sized cells with dark blue cytoplasm, and a high nuclear to cytoplasmic ratio. Cytogenetics were normal (46XX), and RQ-PCR testing for BCR-ABL was negative. She achieved complete remission on a standard ALL protocol, and at time of presentation she was 2 years out from completing treatment [9]. At her last hematology followup, 4 months earlier, she was well with no evidence of relapse. 

 On examination she had no peripheral lymphadenopathy, hepatosplenomegaly, bruising, petechiae, rashes, or signs of anemia. Her abdomen was mildly tender, but there were no palpable masses or signs of peritonitis. Investigations showed a normal total leukocyte count (7.6 × 10^9^/L) and differential, and a normal platelet count (152 × 10^9^/L); however, she had developed a new normocytic anemia (hemoglobin 85 g/L, previously 146 g/L) and had an elevated creatinine of 193 umol/L (CrCl 30.4 mL/min), elevated from her baseline of 51 umol/L. She had a uric acid of 474 umol/L and lactate dehydrogenase of 273 u/L, with normal lipase and amylase levels. Her urinalysis showed microscopic hematuria with no proteinuria. 

**Figure 1:**
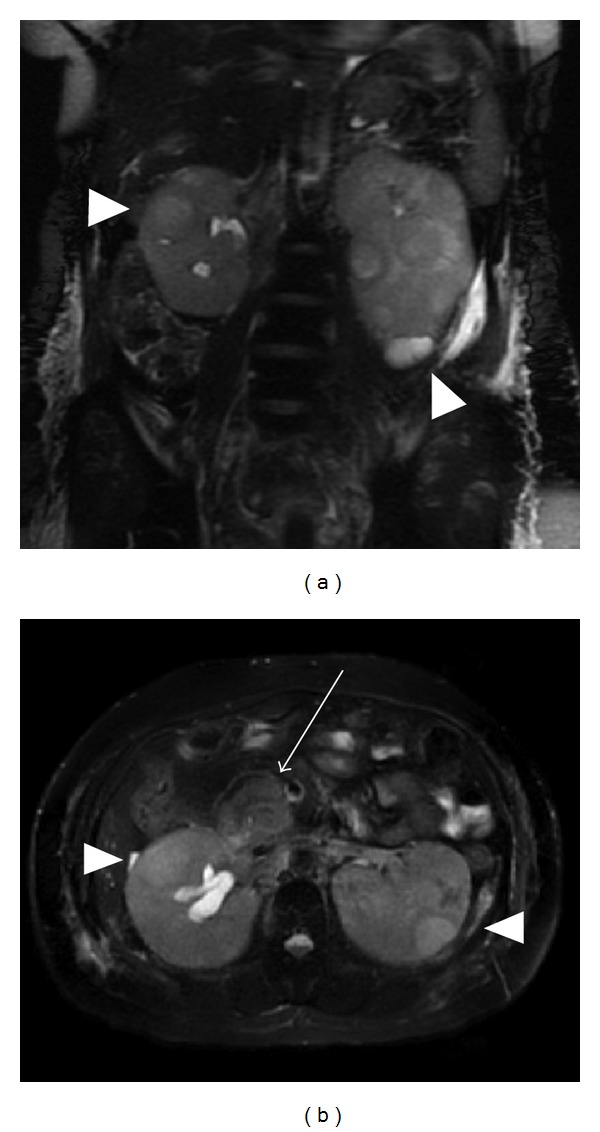
Magnetic resonance imaging of the abdomen showing bilateral renal infiltration. T2-weighted fat-saturated magnetic resonance imaging of the abdomen. (a) Coronal view shows bilateral renal infiltration (arrowheads). (b) Transverse cross-section shows bilateral renal infiltration (arrowheads), unilateral right hydronephrosis, and pancreatic involvement (arrow).

**Figure 2:**
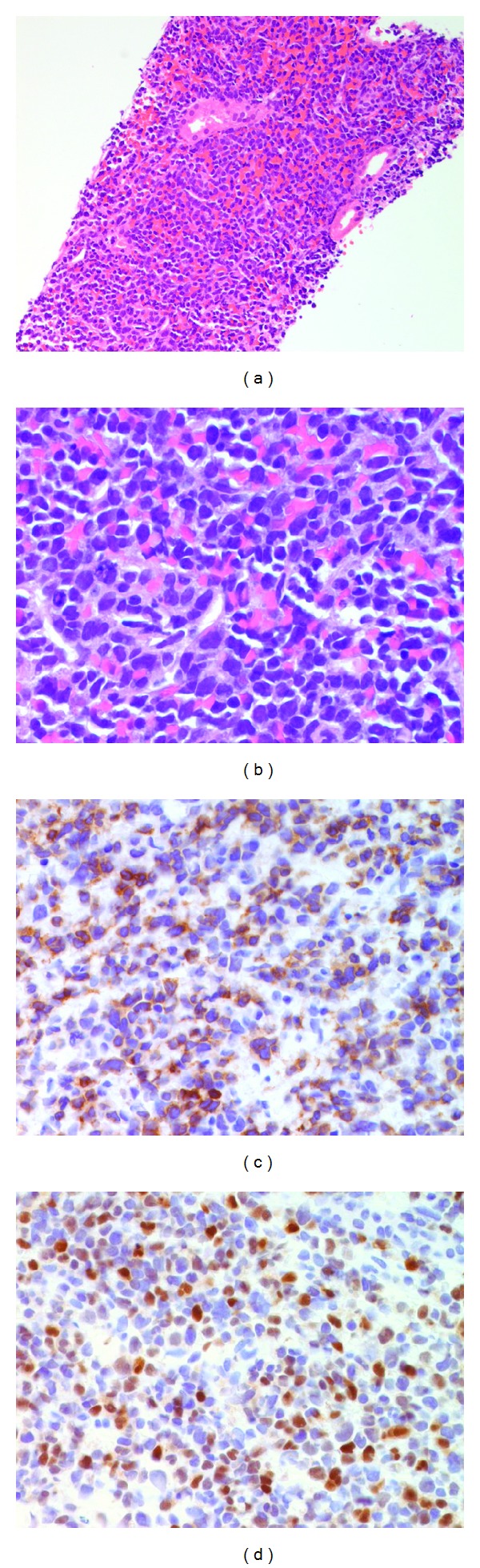
Kidney biopsy showing lymphoblastic infiltration. (a) Histology shows the interstitial lymphoblastic infiltrate surrounding the glomeruli and tubules (×200 magnification). (b) Hematoxylin and eosin stain of the lymphoblasts (×630 magnification). (c) Immunohistochemistry with cells expressing CD79 with cytoplasmic staining (×400 magnification). (d) Immunohistochemistry with cells expressing TdT with nuclear staining (×400 magnification). These findings are typical of lymphoblasts.

 Imaging of the patient's abdomen with ultrasound and noncontrast computed tomography (CT) revealed an enlarged hypoechoic pancreas and bilateral renal enlargement. The kidneys had a diffuse nodular appearance with T2 hyperintensity seen on magnetic resonance imaging (MRI), with an exophytic circumscribed homogeneous left renal mass (5.4 × 4.6 cm) (Figure 1). A biopsy of the kidney mass was performed, and the pathology was consistent with B-cell ALL. The kidney was infiltrated with medium-sized neoplastic lymphoid cells expressing TdT, CD79a, CD43, and CD23 (Figure 2). A subsequent bone marrow biopsy revealed a TdT-positive lymphoblastic population in 5–10% of the bone marrow.

The patient was treated with FLAG (fludarabine, cytarabine, and G-CSF) induction chemotherapy and entered a second complete remission. Her renal function improved but remained less than her baseline (Cr 99 umol/L, CrCl 59.2 mL/min). Her course was complicated by a portal vein thrombosis requiring treatment with anticoagulation. She received 2 more cycles of FLAG as consolidation, with the intent of proceeding to an allogeneic stem cell transplant.

Unfortunately, despite an initial response to therapy, she developed recurrent abdominal symptoms in the days preceding her transplant, and reimaging showed that her extramedullary disease had progressed. In addition to worsening kidney and pancreatic infiltration, a new large retroperitoneal mass that encased the aorta and right psoas muscle (4.5 cm × 3.1 cm × 6.9 cm) was present. The decision was made at that time to treat her symptoms with steroids and abdominal radiation therapy and to pursue purely supportive measures with no further chemotherapy. 

## 3. Discussion

 Extramedullary relapse of ALL to the kidneys is rare and has primarily been reported in the pediatric literature. Kebaili and colleagues described a 13-year-old female with a left solid renal mass discovered on routine ultrasound imaging 32 months after complete remission [8]. Similarly, De and Menell described a 16-year-old male who presented with painless hematuria and a solitary left exophytic renal mass, 36 months after finishing chemotherapy for ALL [10]. In both cases, renal biopsy confirmed lymphoblastic infiltration, while the bone marrow biopsy and aspirate were normal [8, 10]. Rare cases of renal relapse have also been reported in retrospective pediatric studies examining the diagnosis or treatment of extramedullary relapse [11–13]. There was one adult case of extramedullary relapse to the kidney reported in the MRC UKALL12/ECOG 2993 study [14]. Our patient had kidney and pancreatic involvement, but she also had relapsed disease in her bone marrow. Preliminary research suggests that “isolated” extramedullary involvement may, in part, originate from leukemic cells in the bone marrow, even if those cells are not detected by standard laboratory techniques. In one retrospective study, submicroscopic bone marrow involvement was detected via real-time quantitative PCR in the majority of pediatric ALL patients (46/64) who were thought to have isolated extramedullary disease [15]. 

 Clinical presentations of leukemic renal involvement may include renal failure, hypertension, hyperuricemia, and rarely lactic acidosis [16–18]. Acute kidney injury secondary to leukemic infiltration has been seen as the first presentation of ALL, with renal function returning to baseline with treatment [19–21]. Renal failure and high uric acid levels in the setting of a low tumor burden have been described in pediatric patients with isolated renal involvement [17, 22]. Type B lactic acidosis has also been reported, including one case of a 29-year-old man with relapsed T-cell lymphoblastic leukemia isolated to the kidneys [18, 23]. 

 In the adult patient population with ALL, extramedullary relapse has been predominately reported in the postallogeneic stem cell transplantation setting [24]. Two adult cases of posttransplantation leukemic relapse in the kidneys have been reported. Both patients developed worsening renal failure after transplant, and subsequent imaging and biopsy confirmed lymphoblast infiltration of the kidneys bilaterally [25, 26]. While the cause of extramedullary relapse after transplantation is unknown, one hypothesis is a reduced graft versus leukemic effect of the tissue site, with graft cytotoxic T lymphocytes having reduced access to certain “sanctuary” sites [27]. Partial or complete kidney shielding during total body irradiation to prevent radiation nephropathy has also been proposed as a possible mechanism of relapse [27]. 

 There have been few case reports of biopsy-proven pancreatic involvement by ALL either at initial presentation or at relapse. Leukemic infiltration of the pancreas may lead to pancreatitis or biliary obstruction or may remain asymptomatic [21, 28–32]. Based on imaging findings, the assumption was that our patient had pancreatic involvement with ALL in addition to the renal relapse; however, a confirmatory biopsy of the pancreas was not performed. 

 Why is there a predilection of leukemic cells for extramedullary site involvement? The tumor microenvironment is complex and is only beginning to be defined [33]. The chemokine stromal-cell-derived factor-1 (SDF-1) plays an important role in leukocyte trafficking and is constituently expressed in both bone marrow stromal cells and other body organs. Overexpression of SDF-1's associated receptor, CXCR4, on malignant lymphoblasts is associated with extramedullary infiltration in patients with acute leukemia, irrespective of total peripheral blood blast count [34]. Understanding the pathophysiology of the tumor microenvironment creates further avenues for treatment, such as a CXCR4 antibody that is currently in early development for a number of hematological malignancies [35]. 

The prognosis of relapsed ALL in adults is poor, with a median survival of 24 weeks reported in the MRC/EGOG study [14]. Unfortunately our patient's disease progressed despite aggressive chemotherapy, yielding a very low likelihood of a future response from transplantation [1]. Pediatric research has examined the prognosis of extramedullary relapse when it is compared to isolated bone marrow relapse, but it is unclear if these results can be applied to adult cases [2, 36].

## 4. Conclusion

Extramedullary relapse in acute lymphoblastic leukemia is uncommon, with involvement of the kidneys or pancreas being extremely rare. Imaging investigations should be completed, if a patient in remission develops symptoms or signs of renal or pancreatic disease, to investigate for relapse. Further research is needed to improve the treatment of extramedullary disease in adults with ALL.
